# Resonance, self-reflection, and preparedness through a web-based intervention for family caregivers of patients with life-threatening illness receiving specialised home care

**DOI:** 10.1017/S1478951524002086

**Published:** 2025-01-21

**Authors:** Cecilia Bauman, Viktoria Wallin, Sandra Doveson, Peter Hudson, Ulrika Kreicbergs, Anette Alvariza

**Affiliations:** 1 Department of Health Care Sciences, Marie Cederschiöld University, Stockholm, Sweden; 2 Department of Nursing Science, Sophiahemmet University, Stockholm, Sweden; 3 Centre for Palliative Care St Vincents & The University of Melbourne, Melbourne, Australia; 4 Family Medicine and Chronic Care, Vrije University, Brussels, Belgium; 5 Great Ormond Street Institute of Child Health, University College London, London, UK; 6 Research and Development Unit/Palliative Care, Stockholms Sjukhem, Stockholm, Sweden

**Keywords:** Palliative care, family caregivers, web-based support, intervention, home care

## Abstract

**Objectives:**

In home-based care for severely ill patients, family caregivers’ contributions are crucial. This study aimed to explore how a web-based psychoeducational intervention influences family caregivers’ experiences in addressing challenges while caring for a patient with life-threatening illnesses during specialized home care.

**Methods:**

This qualitative study undertook semi-structured interviews with family caregivers of patients with life-threatening illness receiving specialized home care. Family caregivers participated in a randomized controlled trial evaluating a psychoeducational intervention delivered through a website. Interviews were performed with 17 family caregivers; 13 spouses, 2 adult children, 1 parent, and 1 sibling, and analyzed using qualitative content analysis.

**Results:**

The results indicate that the intervention resonated with the family caregivers’ situation which gave them comfort and awareness. It inspired self-reflection on the caregiver role that provided new insights and encouraged communication with the patient. The intervention prepared family caregivers for the patient’s progressing illness and death. While preparing was a help for some, others did not feel ready to face this, which led them to avoid parts of the website.

**Significance of results:**

This psychoeducational web-based intervention guided family caregivers as they addressed challenges in caregiving and prepared for the future, and they valued having access to such an intervention. In a time of decreasing healthcare resources, web-based support may be a useful alternative to in-person interventions. It is important to continue developing, evaluating, and implementing web-based interventions to meet the needs of family caregivers.

## Introduction

A family caregiver of a person with life-threatening illness being cared for at home takes on a range of supportive and medical caregiving activities (Matthys et al. 2022), and must also balance the caregiver role with their own lives (Cai et al. 2021). Caregiving may impact negatively on family caregivers, leading to emotional distress and anxiety (Chan and Ng 2022; Soyaslan and Oksuz 2022; Starr et al. 2022), and may affect their physical health (Zavagli et al. 2022) and quality of life (Liu et al. 2020). In addition, studies have demonstrated that family caregivers face a range of challenges and report unmet practical, emotional, social, and spiritual needs (Marco et al. 2022; Wang et al. 2018; Zavagli et al. 2022)

Many family caregivers have reported a preference for interventions delivered via the internet that can be accessed at a convenient time (Coumoundouros et al. 2023). The internet has become a natural platform for distributing information, is easily accessible, and can diminish geographic and time barriers (Scott et al. 2019; Tieman et al. 2023). Additionally, interventions delivered in person may not appeal to all family caregivers (Holm et al. 2015a). Systematic reviews have shown that web-based interventions may have positive effects on family caregiver outcomes, such as increased self-esteem, self-efficacy, and well-being, as well as decreased levels of strain, anxiety, depression, and distress (Hu et al. 2015; Ploeg et al. 2018; Sherifali et al. 2018), and are useful, safe, and acceptable in palliative care (Finucane et al. 2021).

More knowledge is needed about how web-based interventions can be helpful for family caregivers in their caregiving role. For evaluation guidance, the UK Medical Research Council’s 4-phase framework can be applied (Skivington et al. 2021). This framework emphasizes the importance of using multiple methods in the evaluation design to address a broad range of questions concerning the mechanisms underlying an intervention’s effectiveness. Qualitative studies are particularly valuable for understanding how an intervention can lead to change. This study, therefore, aimed to explore how a web-based psychoeducational intervention influences family caregivers’ experiences in addressing challenges while caring for a patient with life-threatening illnesses during specialized home care.

## Methods

### Design

This study is a substudy of a randomized controlled trial (Clinical Trials identifier NCT05785494). The randomized controlled trial included 205 family caregivers (October 2022–December 2023) and evaluated a psychoeducational intervention delivered through the website “narstaende.se.” The intervention aims to promote family caregivers’ preparedness for both caregiving and death. Intervention effects are currently being analyzed and will be presented elsewhere. The present study is an important part of the evaluation phase of the intervention, and employed an exploratory design with a qualitative inductive approach using data from interviews with family caregivers who had participated in the randomized controlled trial. The study adhered to the Declaration of Helsinki and ethical approval was obtained from the Swedish Ethical Review Authority, approval numbers 2022-02218-02 and 2022-06623-02.

### The psychoeducational web-based intervention

The intervention content is based on evidence from empirical research (Hauksdóttir et al. 2010; Holm et al. 2016; Valdimarsdóttir et al. 2003) and guided by the theoretical framework of Andershed and Ternestedt (2001). This framework illustrates family caregivers’ involvement in care through 3 principal needs: *knowing, being*, and *doing. Knowing* represents family caregivers’ search for knowledge, *being* includes the emotional aspects of spending time together with the patient, and *doing* includes the practical aspects of doing things for the patient. The intervention is delivered through a website featuring short videos that present actors portraying family caregivers as they interact with healthcare professionals. The content addresses issues known to be important for family caregivers and the videos are supplemented by informative texts, a moderated chat forum, and web links. The content is organized into 3 main domains: *Support for you – being a family caregiver* which covers aspects of coping with being a caregiver, *How to give support* which covers emotional and practical aspects of providing care, and *Talk about it* which covers communication and the future. In total, the intervention provides 23 videos, ranging from 2.5 to 8.5 minutes in length, all with descriptive titles that enable family caregivers to choose topics of interest (Table 1). Feasibility testing of the intervention has demonstrated acceptability and usability (Tibell et al. 2022).

**Table 1.** The topics that are addressed on the intervention website “narstaende.se”

Support for you – being a family caregiver	How to give support	Talk about it
Many different emotions	Being a family member and a family caregiver	Thoughts when terminating active illness treatment
About self-care and acceptance	Reflections on the shifting roles	Feelings that may arise when active illness treatment is terminated
Ask for help	Relieve the bad and find the good	Withdrawal of treatment
About family caregivers’ need for help and support from others	Common symptoms, medications, and side effects	Reflections about that quality of life may be improved when the treatment of the disease has side-effects
Concerns about the future	Nutrition and weight	Nice to have talked about
To share thoughts and worries	Advice on coping with eating difficulties	Reflections about what one would want to say with limited time left
May I be happy	Appetite loss	Talk about dying
Acceptance of conflicting feelings	About appetite loss and strategies around mealtimes	About the anxiety and conflicting thoughts that may arise about death
Meeting others	Pain relief	The loss
How illness can change relationships	The importance of pain relief and a discussion about concerns about side effects	Common thoughts and feelings after death of a close person
Continue working or not	Fatigue and rest	When death is closing in
About continuing working or being more at home to spend more time together	Strategies to cope with fatigue	Signs and symptoms that might indicate that life is coming to an end
Finding energy	Little helpful things	Funeral/memorial ceremonies
What might contribute energy and strength	About planning activities and how to give soft tissue massage	How ceremonies can be meaningful in processing the situation
Being me		
About the importance of having moments alone or to meet others		
Breathing exercise		
A breathing exercise for rest and recuperation		

### Setting, inclusion criteria, and procedure

**Figure 1.**
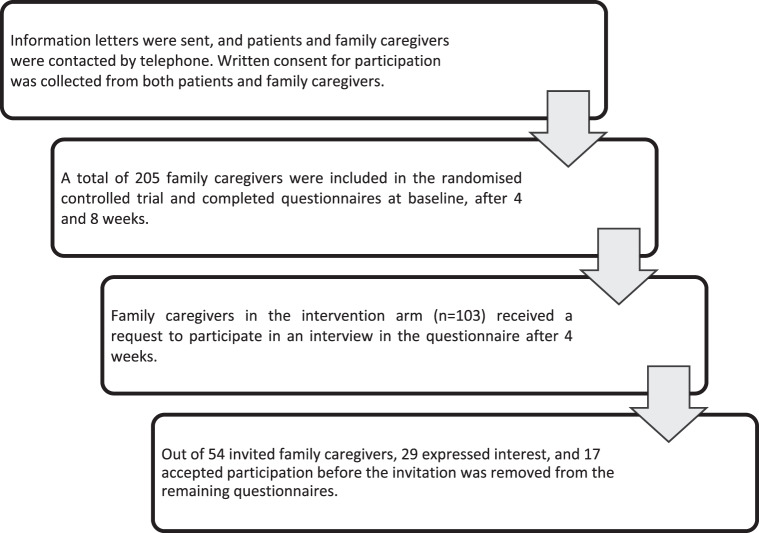
Overview of the inclusion procedure.

The recruitment base for the randomized controlled trial was 5 specialized home care services in a metropolitan area in Sweden, in which multi-professional teams provide round-the-clock home care. The head of each service granted permission for eligible family caregivers to be identified through patients’ records. Inclusion criteria required participants to be a family caregiver (≥18 years) of a patient (≥18 years) with life-threatening illness and palliative care needs, receiving specialized home care. Family caregivers also needed to be able to communicate in Swedish. A letter providing information about the study was sent to the patient together with a letter to the family caregiver. About a week later, patients and family caregivers were contacted by telephone to receive verbal information about the study. Patients who approved their family caregivers’ participation and the retrieval of information about their age and diagnosis from the patient record provided written consent. Family caregivers provided written consent in connection with the data collection. Those who agreed to participate were requested to complete questionnaires at 3 time points (baseline, after 4 weeks, and after 8 weeks). The questionnaire sent after 4 weeks to family caregivers allocated to the intervention arm of the randomized controlled trial included a request to participate in an interview. A total of 54 family caregivers were invited to participate, and 29 expressed interest. Ultimately, 17 family caregivers accepted the invitation, as some were unable to proceed due to factors such as patient deterioration, caregiver burden, and lack of time. These 17 family caregivers were consecutively included in the present study as questionnaires were completed. Sample size was continuously assessed based on the richness of data in relation to the study aim (Malterud et al. 2016). Inclusion was ended when data were considered sufficiently rich to reflect variations in experiences. Consequently, the invitation to participate in an interview was excluded from the remaining questionnaires (Fig. 1).

### Data collection

Data for the present study were collected between January and November 2023 through individual qualitative semi-structured interviews conducted by the first author. Participants could choose either face-to-face interviews (11), telephone interviews (5), or interviews via video conference software (1). A study-specific interview guide with open-ended questions that had been developed by the authors was used. The guide included questions such as “What has been your experience of using the website?,” “What significance has the intervention had for you in your role as a caregiver?,” “How has the intervention helped you prepare for the future?” and “In what way has the intervention been important for you in communicating with the ill person about their illness?” The interviews lasted between 31 and 98 minutes. All interviews were audio-recorded, and field notes of contextual information were written shortly after each interview.

### Data analysis

Data were analyzed using qualitative content analysis as described by Graneheim and Lundman (Graneheim et al. 2017; Graneheim and Lundman 2004; Lindgren et al. 2020). This method was used to describe variations of experiences and to make the voices of the participants heard. Leaning on the method’s epistemological contribution, the analysis developed from descriptive to interpretive (Lindgren et al. 2020). The audio recordings were transcribed verbatim. The first author listened to the recordings repeatedly while reading the transcribed text to validate the transcripts and become immersed in the data. Meaning units, parts of the text pertaining to the study aim and reflecting various experiences regarding the intervention, were extracted by the first author. Each meaning unit consisted of several sentences to preserve its context. With the study aim in mind, descriptive codes were assigned to the meaning units and continually adjusted to closely reflect the contextual meaning of the original text. Codes were kept rich to facilitate contextual understanding and were sorted into categories based on similarities. The first author performed the initial sorting of the codes, which was verified by the last author. Through interpretation of the underlying meaning, categories were formed into themes based on how they interrelated. The themes were refined until considered rich enough to reflect a variation of experiences, a process discussed among all the authors and finalized by the first and last authors. Thematic saturation was carefully considered to avoid themes that were too general and unspecific. To ensure understanding, validate the original context, and prevent a fragmented result, there was repeated movement between the parts and the whole of the text throughout each phase of the analysis process.

## Results

### Participants

In total, 17 family caregivers participated: 12 women and 5 men, with a mean age of 67 years (range 42–85 years). Thirteen were spouses, two were adult children, one a parent, and one a sibling. Their employment status was employed (*n* = 9) and retired (*n* = 8). All were actively involved in the care of the patient and performed various caregiving tasks daily. The patients had been diagnosed with a life-threatening illness a median of 2 years earlier. One patient had an advanced lung disease, while all other patients had advanced cancer. The patients’ mean age was 69 years (range 36–85 years).

The findings illustrate 3 themes that describe how “narstaende.se” influenced family caregivers as they addressed challenges as a caregiver to a patient with life-threatening illness: (1) *Resonates with one’s situation*, (2) *Inspires self-reflection on the caregiver role*, and (3) *Prepares for progressing illness and death*.

### Resonates with one’s situation

The web-based intervention gave family caregivers a feeling that it mirrored their situation, as the topics addressed often felt familiar. They expressed that the scenarios portrayed in the videos clearly resonated with their experiences as caregivers. This helped them become aware of their needs and was described as an important perspective, acknowledged as a rare experience. Others, however, expressed that it did not help to identify themselves with the family caregivers in the videos, since they would have preferred to meet and talk to others in a similar situation:

I’d like to talk to someone whose mother is also ill, and somehow find some kind of friend.

(Family caregiver 1, female).

However, overall, that the situations in the videos resonated with one’s situation was described as helpful, leading to a comfort and inner calm, as illustrated by a family caregiver:

I’ve turned to the website when I’ve felt particularly lonely, really low, and just needing support. It’s been helpful during those times, allowing myself to cry and truly feel how things are because sometimes it can be tough. Identification and comfort are what I would call it. I feel sad when I see something that resembles my situation, at the same time, I find it comforting too.

(Family caregiver 17, female).

Family caregivers described how the web-based intervention reinforced a sense of normalcy in their feelings and experiences because it was built on the shared experiences of others who had gone through similar situations. Such feelings of togetherness were regarded by the family caregivers as comforting, making their challenges feel less daunting. The videos made family caregivers realize how alone they felt, which they had not wanted to admit before. They expressed feeling a connection with the family caregivers in the videos, described as comforting and providing them with a sense of belonging.

### Inspires self-reflection on the caregiver role

Family caregivers described how the web-based intervention provided stability in a situation characterized by worry and insecurity. They found the videos helpful in initiating self-reflection about the caregiver role. Despite the difficulty of this process, they considered it important and supportive. Furthermore, family caregivers described the intervention as a rare source of direction through the complexities of caregiving. This direction inspired valuable self-reflection, helping family caregivers navigate challenges, such as when to encourage the patient or when to step in, and contributed to an increased feeling of being capable in their role. This was explained by a participant as follows:

The uncertainty of whether you’re doing things right or wrong, and realizing you probably can’t do anything wrong, only do your best. So, [the intervention] has made me feel like I’m not doing that badly, perhaps it’s enough sometimes to just be there and to try.

(Family caregiver 12, female).

Family caregivers experienced that the intervention was helpful by stimulating family caregivers’ self-reflection on how to respond to challenging caregiving situations. Family caregivers acknowledged a gap between how they and the patient approached the illness, and the intervention provided reflection on both perspectives. This reflection gave insights that helped family caregivers in how to adapt their approach to caregiving, for example by realizing that spending more time together, instead of focusing on practical matters, could be what the patient valued most. Family caregivers experienced that this self-reflection helped them shift perspectives and alleviated some of their frustration when responding to certain caregiving situations.

A key reason family caregivers wanted to participate in the intervention was to acquire more insights into how to make things easier for the patient. Consequently, family caregivers hoped that the insights gained from self-reflection while using the intervention website would ultimately benefit the patient, as expressed by a man caring for his wife:

I have the videos [on the intervention website] in my head … The more I learn, the better I grasp the situation, learn about her illness, and any advice I can gather on how to support her, anything that helps me become a better caregiver, is good for her.

(Family caregiver 3, male).

Family caregivers experienced the web-based intervention as a source for self-reflection that they knew they could engage with at their own pace over time. They appreciated how the intervention website offered a private space where they could tend to their needs and use for reflection. Since caregiving was so demanding, they said it was a relief to have access to web-based support instead of needing to travel somewhere. However, they also stated that the web-based format could not sufficiently meet their needs for direct contact with other family caregivers nor for immediate responses and tailored advice on specific caregiving issues. Additionally, some family caregivers experienced that the intervention predominantly focused on emotional support and lacked sufficient practical information to fully meet their needs. One participant said:

My need for support is different, I think, from what you’re trying to address. Mostly, I’ve just been looking for information, so I’ve been using regular search engines.

(Family caregiver 9, male).

Through the self-reflection prompted by the intervention, family caregivers described gaining the courage to initiate conversations or consider doing so, about their own experiences and feelings with the patient. This helped them reflect on issues which they felt were difficult and had not previously been raised. One participant said:

I’ve tried to talk to him [the patient] about what I’ve read [on the intervention website], so I think we have discussed things.

(Family caregiver 7, female).

Since they had become more aware of their feelings and experiences, family caregivers described how they had begun to acknowledge painful, yet urgent, issues to discuss with the patient. Consequently, the intervention was described as providing a helpful push to address matters that had been postponed.

### Prepares for progressing illness and death

Family caregivers described how the intervention enabled them to reflect more actively on the future and was considered helpful in trying to prepare. They appreciated the videos addressing the end of life even though they were painful to watch. Watching these videos provided an opportunity to process their thoughts and prepare for facing the patient’s end of life. They described being aware that the progression of the patient’s illness and impending death were inevitable; however, preparing for it was difficult. Family caregivers described how the videos had helped them to find the courage to start thinking ahead. The intervention also allowed them to address their concerns more privately, since actively taking measures to prepare could be experienced as inappropriate, causing family caregivers to feel ashamed or disloyal toward the patient. This was exemplified by a family caregiver who said:

I started googling, trying to figure out how a funeral works. I almost felt ashamed, like, why am I even doing this? Asking someone is an even higher threshold. That there’s a website where you can just click around and take a look, I can see the benefit in that … The website is both helpful and challenging, making me enter a dark place I will perhaps eventually need to step into.

(Family caregiver 6, male).

Family caregivers described having access to the intervention website as positive, even though they did not necessarily want to use all the content available at a given point in time. They believed they would use the parts of the website that addressed the future at a time closer to the patient’s end of life when they needed to prepare for impending death. Other participants conveyed how some parts of the intervention website could help them process the caregiving period after the patient’s death. Family caregivers expected to have more time then and feel a greater need to reflect on their experiences, as exemplified by a participant:

Later on I could go in and take a look [at the intervention website], like, “this is what went down” and “that’s how it happened.” In that way, maybe I can start grasping it all. At that stage, I’ll have a lot more time to sit back, think it over, and process it.

(Family caregiver 8, male).

Although they knew that the patient would eventually die, not all family caregivers felt ready to use the intervention website to prepare for this. They described how thoughts about the future when using the intervention felt overwhelming, like facing an emotional darkness they were not ready for. While they intentionally avoided those parts of the intervention website, they also acknowledged that this might leave them unprepared. Family caregivers described how they deliberately postponed preparing for the future as a way of bearing the emotional challenge and shielding themselves from the patient’s inevitable illness progression and death. For some, everyday caregiving responsibilities were so consuming, they did not have the emotional capacity left to use the website to prepare for the future. Some also described how they prepared for the future in ways other than those suggested on the website or that the support offered by the intervention was limited because the patient was not that ill, or because the family caregivers felt they were already sufficiently prepared.

## Discussion

The findings indicate that the participating family caregivers experienced that the web-based intervention “narstaende.se” helped them address challenges since the videos on the website resonated with situations from their lives as caregivers, which provided comfort. The website inspired self-reflection on the caregiver role by providing new insights and encouraging the initiation of conversations with the patient about their experiences and feelings. The web-based intervention also helped family caregivers prepare for the patient’s progressing illness and impending death; however, not all family caregivers felt ready to face this.

The family caregivers conveyed how the web-based intervention provided comfort by resonating with caregiving situations familiar to them. This is comparable to Washington et al. (2021) who found that feelings of comfort may come from informal support through shared firsthand caring experiences. The intervention in the present study acknowledged the participants’ situation, promoted feelings of togetherness, and neutralized challenges. Evoked feelings of togetherness align with the findings of Daynes-Kearney and Gallagher (2024) regarding online support. They found that shared experiences and the acknowledgment of not being alone fostered a sense of community, even for those only using the online support as observers.

Through the web-based intervention in the present study, family caregivers were inspired to self-reflect on the caregiver role which gave new insights, such as how to respond to certain caregiving situations. This may be of importance since earlier studies have revealed family caregivers’ uncertainties in balancing caregiving demands in their everyday lives (Eriksson et al. 2019; Nissim et al. 2017). These studies show that family caregivers seem to find it particularly challenging to learn how to manage new and unfamiliar illness-related issues and to ensure the well-being of the patient, as was also confirmed by the participants in the present study. If support from the healthcare system is lacking, family caregivers may feel left to solve complex caregiving challenges on their own (Banadinović et al. 2023).

The present study reveals dual perspectives among family caregivers. On the one hand they acknowledged that the intervention helped them to prepare, and on the other hand they deliberately avoided preparation as a means of distancing themselves from the patient’s impending death. This is also confirmed by Zhang et al. (2023) who suggest that both avoidance and actively preparing for the patient’s death are strategies family caregivers use during the caregiving period. Breen et al. (2018) contribute to a further understanding, showing that family caregivers of patients in need of palliative care attempt to prepare for the patient’s death while experiencing complex feelings of hope and fear for the future.

In summary, the findings of this study suggest that family caregivers’ value having access to web-based support that helps them address challenges in caregiving and prepare for the future. It is reasonable to assume that this could also promote preparedness for both caregiving and death. Preparedness among family caregivers of older adults was recently conceptualized (Dal Pizzol et al. 2023) by 4 core attributes: self-confidence, having knowledge, handling emotions, and being developed over time. These attributes are apparent in the results of the present study and congruent with the framework of Andershed and Ternestedt (2001), which underpins the intervention and highlights the significance of family caregivers’ involvement in care through knowing, being, and doing. Preparedness for caregiving has, in the context of palliative care, been described as an ongoing process, including becoming aware of the seriousness of the illness, adjusting to a changing situation, and anticipating the future (Holm et al. 2015b). Preparing to care for a close person with life-threatening illness may, therefore, also mean preparing for that person’s impending death (Häger Tibell et al. 2023; Janze and Henriksson 2014). Preparedness can be acquired through information from programs and resources tailored to family caregivers’ needs (Dal Pizzol et al. 2023). This gives prominence to web-based interventions as a resource to promote family caregiver preparedness.

The strengths of this study include a sufficient number of participants with variation regarding age and relationship to the patient. Adherence to the participants’ preferences for face-to-face or telephone/digital interviews may have facilitated participation. There was no difference in the length or richness of the interviews based on the interview method. The analysis process was transparent and rigorous, resulting in varied descriptions without favoring positive experiences, even though these were mostly expressed. The study is further strengthened by the fact that the intervention was theory-based, which confirms the results. However, certain limitations should be acknowledged. No family caregivers from cultures other than traditionally Swedish participated in this study, which is unrepresentative of the current Swedish population, potentially limiting the study’s trustworthiness. It could also be considered a limitation that the demographic data describing the participants were limited. Additionally, it cannot be ruled out that family caregivers with predominantly positive experiences chose to participate in the study, which might have influenced the results.

## Conclusion

The web-based intervention, “narstaende.se” provided family caregivers with guidance in caregiving through resonance, self-reflection, and preparedness. However, some family caregivers experienced the guidance from the intervention as limited, since it did not provide enough contact with others, practical information, or immediate responses, or they did not feel ready to face all the website’s content. These are important findings that could guide future development of effective web-based interventions. Continued development, evaluation, and implementation of such interventions are essential to meet the needs of family caregivers. This study contributes to the field by raising the voices and experiences of family caregivers as the primary recipients of the intervention.
